# An Ultrasensitive Long-Period Fiber Grating-Based Refractive Index Sensor with Long Wavelengths

**DOI:** 10.3390/s16122205

**Published:** 2016-12-21

**Authors:** Qiu-Shun Li, Xu-Lin Zhang, Jian-Guo Shi, Dong Xiang, Lan Zheng, Yan Yang, Jun-Hui Yang, Dong Feng, Wen-Fei Dong

**Affiliations:** 1 Key Biosensor Laboratory of Shandong Province, Biology Institute of Shandong Academy of Sciences, Jinan 250014, China; shijg@sdas.org (J.-G.S.); zhengl@sdas.org (L.Z.); swdz2@sdas.org (Y.Y.); swdz@sdas.org (J.-H.Y.); shenghua@sdas.org (D.F.); 2 State Key Laboratory on Integrated Optoelectronics, College of Electronic Science and Engineering, Jilin University, Changchun 130012, China; xulin_zhang@jlu.edu.cn; 3 CAS Key Laboratory of Bio-Medical Diagnostics, Suzhou Institute of Biomedical Engineering and Technology, Chinese Academy of Sciences, Suzhou 215163, China; xd789@mail.ustc.edu.cn

**Keywords:** long-period fiber grating, refractive index, sensor, transmission spectrum, long wavelength

## Abstract

The response of a novel long-period fiber grating (LPFG) with a period of 180 µm to a surrounding refractive index (RI) was investigated. The results displayed that, with the increase in RI of the surrounding media of cladding glass in the grating region, the resonant peak located at 1336.4 nm in the transmission spectrum gradually shifts towards a shorter wavelength, while the resonant peak located at 1618 nm gradually shifted towards a longer wavelength. Moreover, the resonant peak at 1618 nm is much more sensitive to the surrounding RI than that of the one at 1336.4 nm. Compared with the conventional LPFG and other types of wavelength-interrogated RI sensors, such as ring resonators, surface plasmon resonance sensors, and Fabry–Perot interferometric sensors, this novel LPFG possesses a higher sensitivity, which achieved 10,792.45 nm/RIU (RI unit) over a RI range of 1.4436–1.4489.

## 1. Introduction

Long-period fiber gratings (LPFGs) are a class of gratings that typically have a period on the level of hundreds of micrometers and can couple the fundamental mode with co-propagating modes [1,2]. A prominent feature of LPFGs is that they are very sensitive to changes in the refractive index (RI) of the surrounding environmental media on the cladding glass surface in the grating region, which makes LPFGs well suited to monitor the surrounding RI. LPFGs have the following advantages in RI sensing aspects: (1) they have ultra low back-reflection loss and low insertion loss; (2) measuring liquid concentration and RI is very simple and convenient due to their small size; (3) the sensing signal belongs to the wavelength interrogation, avoiding the influence of fluctuation and loss of light intensity; (4) the entire all-optical fiber sensing system including the LPFG can not only realize an online, fast, and accurate determination to RI of the usual environmental media in real time, but can also be used to measure the RI in harsh environments and special occasions; (5) and they can be utilized to implement the remote telemetry for RI.

At present, as an RI sensor, the LPFG has been used to monitor ethylene glycol concentration [3], dimethylsulfoxide [4], olive oil [5], chloride ion concentration [6], *Escherichia coli* bacteria [7], organic aromatic compounds [8], hydrocarbons [9], etc. However, in order to exploit and extend the application in the biological and chemical molecular detection, the sensitivity of LPFGs still needs to be further improved.

In this paper, an extremely sensitive RI sensor is presented by employing LPFGs to measure air, water, and different concentrations of glycerol solutions. The results suggest that, with increasing RI of surrounding media, the two resonant peaks of the attenuation bands in the transmission spectrum gradually blue-shift and red-shift, respectively. The sensitivity of the right resonant peak is much more than that of the left resonant peak. In addition, a sensitivity of 10,792.45 nm/RIU (RI unit) was achieved by the right resonant peak over a RI range of 1.4436–1.4489, which is much higher than that of other wavelength-interrogated RI sensors.

## 2. Fabrication and Testing of the LPFG

### 2.1. Fabrication of the LPFG

In this experiment, the silica single-mode optical fiber (Corning SMF-28), which was purchased from Corning Incorporated Company, Corning, NY, USA, has a core diameter of 8.25 μm and a cladding diameter of 125 μm in order to fabricate a LPFG. Prior to fabricating the LPFG, the fiber was hydrogen-loaded for more than a month at room temperature and 100 atm, which increased the photosensitivity of the fiber. Then, the grating was generated by exposing the fiber to an UV KrF excimer laser (Lumonics PM886) with a wavelength of 248 nm through an amplitude mask with a period *Λ* = 180 μm and a total length *L* = 45 mm. In order to fabricate a highly sensitive LPFG, the UV laser pulse energy was set to 300 mJ, and each single exposure time was adjusted to 60 s. Subsequently, the LPFG was annealed for 10 h at 80 °C for stabilization.

### 2.2. The Testing of the LPFG

Acetone, 2-propanol, methanol, and glycerol were purchased from Shanghai Sinopharm Chemical Reagent Co., Ltd., Shanghai, China.

Since LPFGs are extremely susceptible to strain and bend, a proper polytetrafluoroethylene holder was firstly designed to fix it without changing its tension and bending state. The LPFG was further immersed in a mixture of 2-propanol, methanol, and acetone with a volume ratio of 1:1:5 for approximately 15 min at room temperature to remove the surface organics, followed by rinsing with abundant deionized water. Then, the LPFG was immersed into a kind of glycerol aqueous solution for 3–5 min. Subsequently, the glycerol aqueous was drawn out and the LPFG was rinsed with Millipore deionized water several times. After that, the next measurement was carried out. The transmission spectra of the LPFG were recorded using an optical spectrum analyzer (OSA, yokogawa, AQ6375B), which offered a measurement wavelength range from 1200 nm to 2400 nm, a resolution of 0.05 nm, and a minimum integration time of 1 ms. To minimize or avoid wavelength shifts induced by a change of temperature, all measurements were carried out at 15 (±0.1) °C in a clean laboratory room.

## 3. Results and Discussion

### 3.1. The Sensing Principle of the LPFG to the RI

The RI sensing based on the LPFG utilizes the coupling characteristics between the core mode and the cladding mode of the LPFG. When the external RI is changed, the coupling of the fundamental mode and the cladding mode is affected. It is well known that, for LPFGs, the relationship between period *Λ* and resonant wavelength *λ_res_* can be obtained simply by the mode-matched equation as follows [10,11,12]:

(1) $$\lambda_{res}=(n_{co}^{eff}-n_{cl,m}^{eff})\Lambda \;m\;=\;1,2,3,\;...$$

where $n_{co}^{eff}$ and $n_{cl,m}^{eff}$ are the effective RI of the guided core mode and the *m*-th cladding mode, respectively. *λ_res_* is the coupling wavelength, and Λ is the period of the LPFG.

From Equation (1), it can be seen that the effective RI of the cladding mode will vary when the RI of the ambient environment media around the grating region changes, which induces the shift of the resonant wavelength and alterations of the resonant intensity in the LPFG transmission spectra. According to the change in the transmission spectra, the RI or the concentration of the substance can be deduced.

### 3.2. Response of the LPFG to the Surrounding Media

**Figure 1:**
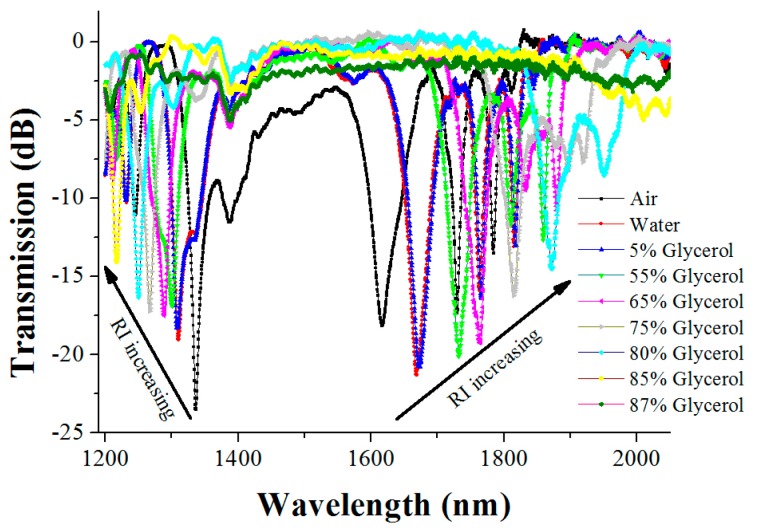
The transmission spectra of the long-period fiber grating (LPFG) in different environment media.

In order to study the sensitivity of the as-fabricated LPFG to the RI, we chose air, pure water, and different concentrations of the glycerol solution as the environment media with the different RI and tested the responses of the LPFG to them. The transmission spectra of the LPFG in the different media are given in Figure 1. As can be seen from Figure 1, when the surrounding medium of the grating is successively varied from air to water, a 5% glycerol solution, a 55% glycerol solution, a 65% glycerol solution, a 75% glycerol solution, an 80% glycerol solution, and an 85% glycerol solution, the resonant peak located at 1336.4 nm is gradually blue-shifted (shift towards a shorter wavelength), while the resonant peak at 1617.2 nm is gradually red-shifted (shift towards a longer wavelength).

**Figure 2:**
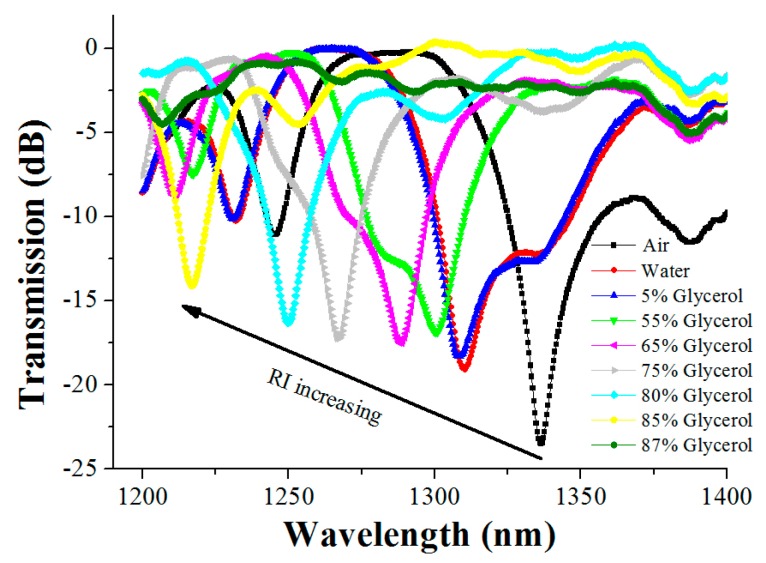
The transmission spectra of the LPFG in different environment media over a wavelength range from 1200 nm to 1400 nm.

**Figure 3:**
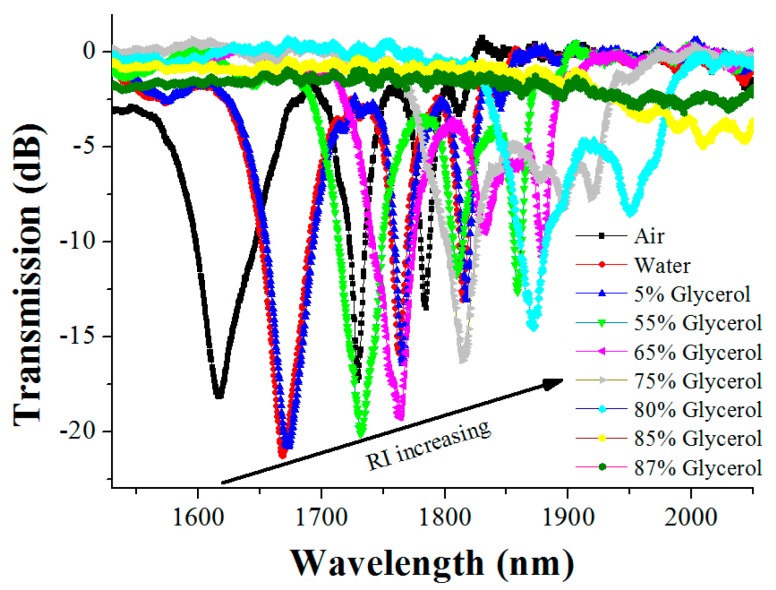
The transmission spectra of the LPFG in different environment media over the wavelength range from 1530 nm to 2050 nm.

In order to more clearly observe the variation of the resonant wavelength, the amplificatory figures of the above two regions are further given in Figure 2 and Figure 3, respectively. Figure 3 indicates that, as the surrounding medium changes from air to an 80% glycerol solution, the right resonant peak (at a wavelength of 1617.2 nm) has a red shift of 254.8 nm; nevertheless, the left resonant peak (at a wavelength of 1336.4 nm) has a blue shift of only 86.4 nm, as shown in Figure 2. Thus, in the above RI range, the shift of the right resonant peak is much more 168.4 nm than that of the left one, that is, the shift distance of the right resonant peak is 2.95 times that of the left one, which means that the right resonant peak is more sensitive to the RI than the left resonant peak.

The sensitivity of LPFG to RI can be described by Δ*λ_res_*/Δ*n* (nm/RIU) [13]. The RI sensitivities of the two resonant peaks in the transmission spectra of this LPFG in the different RI ranges are listed in Table 1. As shown in Table 1, with the increase in the surrounding medium RI, the sensitivities of the two resonant peaks for this LPFG are all increased. Over the RI range of 1.4436–1.4489, the RI sensitivity of the right resonant wavelength is up to 10,792.45 nm/RIU. Within the RI scope of 1.4489–1.4552, the RI sensitivity of the left resonant wavelength reaches over 5269.84 nm/RIU. When the normal LPFG has a poor sensitivity, for instance, the sensitivity of the LPFG is merely 40 nm/RIU in the literature presented by Rego and coworkers [14]. Even when the RI of the environmental media is close to that of the cladding, the RI sensitivity is generally only a few hundred nanometers per unit of RI. For example, in the previous literature [15], the most sensitive RI region for the etched LPFG was 1.400–1.453. The maximum sensitivity is about ~789.47 nm/RIU. Consequently, compared with the conventional LPFG, the RI sensitivity of this LPFG was remarkably enhanced.

Interestingly, even in the RI range 1–1.3334, the LPFG also has a very high sensitivity. As illustrated in Table 1, the difference in the resonant wavelength between air and water for the left resonant peak and the right resonant peak reaches 26 nm and 51.6 nm, respectively. In the RI range 1–1.3334, the RI sensitivity of the left resonant peak is approximately equal to 77.98 nm/RIU, while it is about 154.77 nm/RIU for the right resonant peak. In general, between air and water, the resonant wavelength of the LPFG merely has a difference of a few nanometers [16]. This indicates that, compared with the conventional LPFG, the sensitivity of this LPFG is increased by a dozen times in the RI range 1–1.3334.

It is worth noting that the RI sensitivity of the right resonant peak is much higher than that of the left resonant peak over the same RI range. As listed in Table 1, in the RI ranges of 1–1.3334, 1.3334–1.3406, 1.3406–1.4139, 1.4139–1.4293, 1.4293–1.4436, and 1.4436–1.4489, the sensitivity of the right resonant peak to RI is 1.98 times, 1.83 times, 8.11 times, 2.47 times, 2.48 times, 3.33 times the left one in sequence.

Another very interesting phenomenon is that, in the 85% glycerol solution, the left resonant peak can still be clearly discerned. However, the right resonant peak is very weak such that it is almost impossible to be identified. In the 87% glycerol solution, the right resonant wavelength is already unrecognizable, and the right transmission spectrum of the LPFG with the right resonant peak is almost a straight line, as displayed in Figure 3. This phenomenon manifests in the fact that, for the right resonant peak, the RI sensitivity is increased, but the measurable RI range is significantly small.

**Figure 4:**
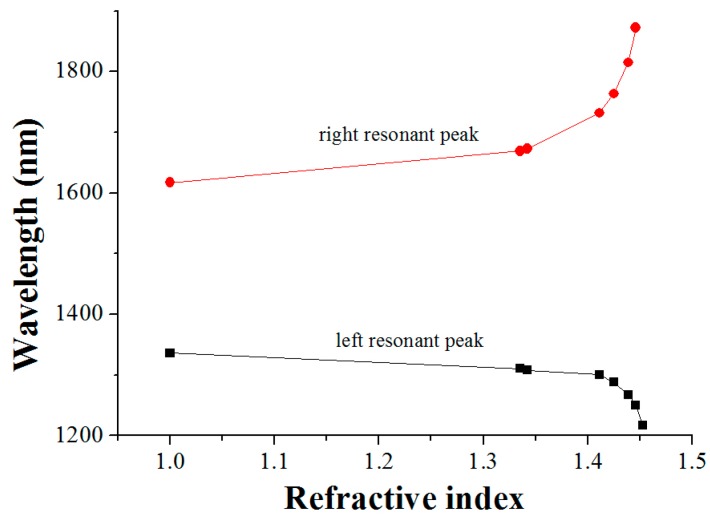
The relationship between the resonant wavelength and RI.

**Figure 5:**
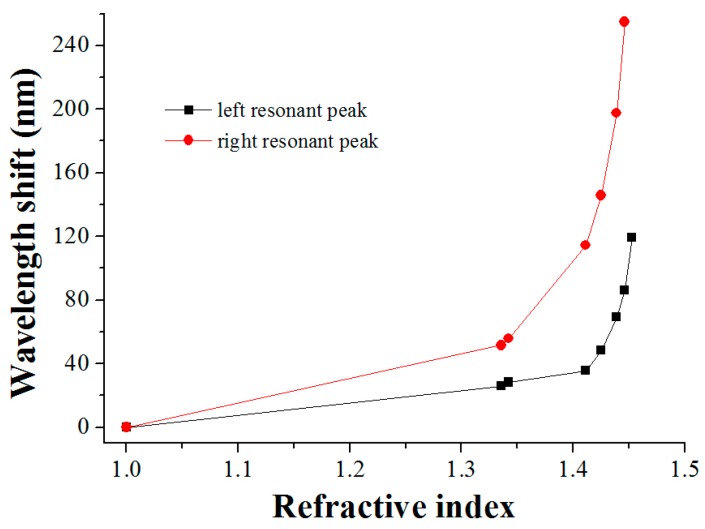
The wavelength changes of the two resonant peaks in the transmission spectra of the LPFG with the increase in RI.

The variation tendency of the two resonant peaks with the increase in RI is exhibited in Figure 4 and Figure 5. As illustrated in Figure 4, the right resonant peak gradually shifts towards a longer wavelength, and the left one towards a shorter wavelength, when the surrounding RI of the grating region increases. Comparing Figure 4 and Figure 5, the two resonant peaks have one thing in common: the RI sensitivity is higher when the environmental RI is closer to that of the cladding. Additionally, when the RI of the measured medium approaches that of the cladding, drastic shifts of the resonant wavelength occur, which is consistent with the performance of the conventional LPFG [16]. It should be pointed out that the ultrasensitive property of the right resonant peak is further distinctly observed in Figure 5. As can be seen in Figure 5, particularly in the RI range of 1.4139–1.4489, the slope of the right resonant wavelength line is nearly close to 90 degrees and the resonant wavelength shift is exceedingly large, which means that the LPFG has an optimal sensitivity in this segment.

### 3.3. Theoretical Analysis and Numerical Simulations

**Figure 6:**
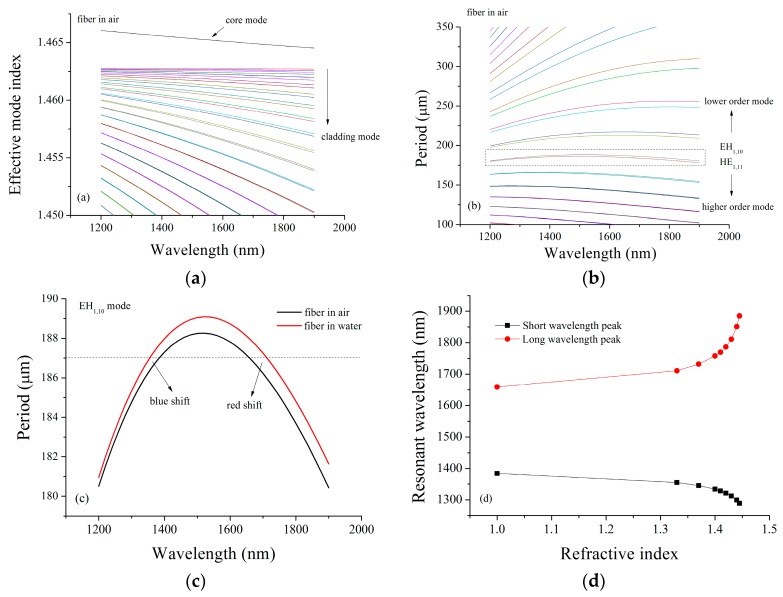
Schematic diagram of theoretical simulation: (**a**) a core mode and a series of cladding modes supported in the fiber; (**b**) the relation between period and order mode; (**c**) the relation between period and wavelength; (**d**) the relation between the resonant wavelength and RI.

To explain the experimental results, we performed a simulation using optical fiber theory [17,18,19]. We first wrote the wave solution in each layer in the form of Bessel functions and matched the boundary conditions to obtain the eigenmode equation. The fiber parameters are chosen as *n_core_* = 1.4681, *n_cladding_* = 1.4628, *r_core_* = 4.15 μm, and *r_cladding_* = 62.5 μm. We first considered the fiber grating exposed in air and calculated the effective mode index of the fiber. The results are shown in Figure 6a, where we find a core mode and a series of cladding modes supported in the fiber. Then, we considered the mode coupling between the core mode and cladding modes and calculated the period using the coupling equation for the LPFG: *Λ* = *λ_res_*/($n_{co}^{eff}$ − $n_{cl,m}^{eff}$). We show the results in Figure 6b. We found that, in the wavelength region of interest, the slope of the curve was positive for the lower order mode, but negative for the higher order mode. Interestingly, for the mode in-betweens such as the EH1,10 and HE1,11 modes highlighted by the dashed box, the change of the lines was not monotonous. We take EH1,10 mode, for instance, and provide a detailed plot in Figure 6c by the black line. It is easy to see that, given a period there, such as the dashed line, there are two resonances due to the nonmonotonic curve. Moreover, the properties for the two resonances are totally different. We considered the fiber grating exposed in water and calculated the relation between the period and the wavelength and show the results in Figure 6c by the red line. It was found that the short wavelength resonance exhibited a blue shift, while the long wavelength resonance exhibited a red shift. It is also noted that the red shift is more sensitive than the blue shift due to the non-symmetry of the curves. Furthermore, we calculated the resonant wavelength for the two resonances as a function of the RI of the surroundings. The results are given in Figure 6d, where the aforementioned distinct properties of the two resonances can be easily observed. We can conclude safely that the red shift mode and blue shift mode in our experiments both resulted from the mode coupling between the core mode and the EH1,10 cladding mode.

In contrast to other types of wavelength-interrogated RI sensors, the RI sensitivity of 10,792.45 nm/RIU of this LPFG sensor is also exceedingly higher. For instance, it is approximately 3.46 times greater than that of the widely used wavelength-interrogated surface plasmon resonance (SPR) sensor (3118.52 nm/RIU) [20]. It is significantly increased by 3.08 times in comparison with the ring resonator sensor (3500 nm/RIU) [21] and about 13.49-fold higher than that of the split ring plasmonic nanostructures sensor (800 nm/RIU) [22].

What is more, the sensitivity and sensitive range of this LPFG sensor to RI can continue to be improved and adjusted by coating nanofilm on the surface of the LPFG [10,15,23,24,25,26]. The kinds of LPFGs with a period of 180 µm and red-shifted resonant peaks are extremely conducive to applications in physical, biological, and chemical sensing due to their overwhelmingly high RI sensitivity.

## 4. Conclusions

An ultrasensitive LPFG sensors with a period of 180 µm is here revealed. The red-shift resonant peak with long wavelengths is more sensitive to RI than the blue-shift resonant peak. Compared with the conventional LPFG, this kind of LPFG has an overwhelmingly higher sensitivity that can reach up to 10,792.45 nm/RIU over a RI range of 1.4436–1.4489. Even if compared to other types of RI sensors, such as the SPR sensor, the nanowire array sensor, and the ring resonator sensor, the LPFG with a red-shifted resonant peak is still more sensitive. The LPFG can be utilized for medical diagnostics, food quality testing, environmental monitoring, biohazard detection, and homeland security.

## Figures and Tables

**Table 1:** The refractive index (RI) sensitivities of the two resonant peaks in the transmission spectra of the LPFG.

Ambient Media	Air and Water	Water and 5% Glycerol Solution	5% Glycerol Solution and 55% Glycerol Solution	55% Glycerol Solution and 65% Glycerol Solution	65% Glycerol Solution and 75% Glycerol Solution	75% Glycerol Solution and 80% Glycerol Solution	80% Glycerol Solution and 85% Glycerol Solution
RI range	1–1.3334	1.3334–1.3406	1.3406–1.4139	1.4139–1.4293	1.4293–1.4436	1.4436–1.4489	1.4489–1.4552
Shift of the left resonant peak (nm)	26	2.4	7.2	12.8	20.8	17.2	33.2
RI sensitivity of the left resonant peak (nm/RIU)	77.98	333.33	98.23	831.17	1454.55	3245.28	5269.84
Shift of the right resonant peak (nm)	51.6	4.4	58.4	31.6	51.6	57.2	-
RI sensitivity of the right resonant peak (nm/RIU)	154.77	611.11	796.73	2051.95	3608.39	10,792.45	-

Notes: Δ*λ_res_* (nm) is denoted as the maximum wavelength difference between two kinds of media; the left resonant peak is denoted as the resonant peak at 1336.4 nm; the right resonant peak is denoted as the resonant peak at 1617.2 nm.
